# Item response theory analysis and properties of decisional conflict scales: findings from two multi-site trials of men with localized prostate cancer

**DOI:** 10.1186/s12911-019-0853-5

**Published:** 2019-07-04

**Authors:** Rachel A. Pozzar, Donna L. Berry, Fangxin Hong

**Affiliations:** 1 Dana-Farber Cancer Institute, 450 Brookline Ave., Boston, MA USA; 2 School of Nursing, Bouvé College of Health Sciences, Northeastern University, 360 Huntington Ave., Boston, MA USA

**Keywords:** Cancer, Conflict, Decision making, Oncology, Prostate, Psychometrics

## Abstract

**Background:**

Decisional conflict is associated with decision quality and may affect decision outcomes. In the health sciences literature, the Decisional Conflict Scale is widely used to measure decisional conflict, yet limited research has described the psychometric properties of the Decisional Conflict Scale subscales and of the low literacy version of the scale. The purpose of this secondary data analysis was therefore to examine properties of the original (DCS-12) and low literacy (LL DCS-10) Decisional Conflict Scales using Classical Measurement Theory and Item Response Theory.

**Methods:**

Data from two multi-site trials of men with prostate cancer were used to analyze the DCS-12, LL DCS-10, and an aggregated DCS-12 dataset in which five response options were aggregated into three. Internal consistency was estimated with Cronbach’s alphas. Subscale correlations were evaluated with Pearson’s correlation coefficient. Item difficulty, item discrimination, and test information were evaluated using Graded Response Modeling (GRM). The likelihood ratio test guided model selection.

**Results:**

Cronbach’s alphas for the total scales and three of four subscales were ≥ 0.85. Alphas ranged from 0.34–0.57 for the support subscales. Subscale correlations ranged from 0.42–0.71 (*P* < 0.001). Items on the DCS-12 exhibited the widest range of difficulty. Two items on the support subscale had low to moderate discrimination and contributed little information. Only the DCS-12 was informative across the full range of decisional conflict values.

**Conclusions:**

Lack of precision in the support subscale raises concerns about subscale validity. The DCS-12 is most capable of discriminating between respondents with high and low decisional conflict. Evaluation of interventions to reduce decisional conflict must consider the above findings.

**Electronic supplementary material:**

The online version of this article (10.1186/s12911-019-0853-5) contains supplementary material, which is available to authorized users.

Decisional conflict is defined as uncertainty about what action to take when a choice is associated with risk or uncertainty [1]. Since the 1990s, research has suggested decisional conflict plays a key role in determining decision quality and may affect decision outcomes. In the clinical context, high decisional conflict is associated with delayed decision making [2], increased regret [3], and decreased quality of life [4]. Individuals who experience high decisional conflict have been found to be more likely to blame their health care provider for negative outcomes associated with a decision [5]. Interventions that aim to improve decision quality often are evaluated based on their ability to reduce decisional conflict. For example, a 2017 Cochrane review of decision aids for people facing health treatment or screening decisions revealed that decisional conflict was an outcome measure in 63 of 105 included studies [6].

In oncology settings there are exemplar situations, notably localized prostate cancer and early stage breast cancer, that embody a high-stakes decision in the reality that no one medical therapeutic approach can be identified as the most efficacious. These preference-sensitive decisions are challenging and difficult for not only the diagnosed individual and family but also the clinician [7]. Given the influence of decisional conflict on decision quality and its role in the evaluation of interventions, there is a need to critically examine the ways in which decisional conflict is measured in the health sciences literature.

The purpose of this paper is to describe current conceptual and operational definitions of decisional conflict and provide an overview of what is known about the psychometric properties of instruments that measure decisional conflict. We provide new evidence regarding the psychometric properties of the original and low literacy versions of the Decisional Conflict Scale [8].

## Background

### Conceptual definitions of decisional conflict

Decisional conflict was first described by psychologists Janis and Mann, who proposed the Conflict Theory Model of Decision Making [9]. The authors asserted that a cause of error in decision making is the individual’s desire to quickly alleviate the stress associated with making a difficult decision. In this context, decisional conflict refers to an individual’s desire to both accept and decline an option. Decisional conflict was established in the nursing lexicon in 1988, when the North American Nursing Diagnosis Association-International (NANDA-I) added decisional conflict to its taxonomy of nursing diagnoses. NANDA-I refined the construct and defined it as “uncertainty about [the] course of action to be taken when [a] choice among competing actions involves risk, loss, or challenge to values and beliefs” (p. 365) [10]. The defining characteristics of and factors related to decisional conflict according to NANDA-I are provided in Additional file 1.

In the mid-1990s, O’Connor expanded the conceptual definition of decisional conflict to include “a state of uncertainty about the course of action to take” (p. 25) [1]. O’Connor and colleagues later proposed a mid-range theory of health decision making known as the Ottawa Decision Support Framework (ODSF) [11]. The ODSF asserts that decisional conflict represents an unresolved need that adversely affects decision quality. The ODSF states that the modifiable factors that contribute to decisional conflict include lack of knowledge, unrealistic expectations of the likelihood of outcomes, unclear values, unclear perceptions of others’ opinions, social pressure to choose one option, lack of support from others, lack of decision-making skills, and lack of other resources [12]. Together, these modifiable factors comprise an operational definition of decisional conflict [1].

### Operational definitions of decisional conflict

The Decisional Conflict Scale (DCS) was first developed by O’Connor in 1995 [1] and is the only known instrument which measures decisional conflict in health and social sciences research. Three versions of the DCS have been developed for use in research, including the original statement format DCS [1], the question format DCS [8], and the low literacy question format DCS [8]. Of these, the original DCS and the low literacy DCS are the versions most widely cited in the literature; as such, the following discussion is limited to these two versions of the scale. Items and subscales of these versions as developed by O’Connor [8] are provided in Additional file 2.

#### Original DCS

The most widely used version of the DCS is a 16-item instrument comprised of five subscales: (1) informed, (2) values clarity, (3) support, (4) uncertainty, and (5) effective decision [1]. The four-item effective decision subscale is only administered to individuals who have made the decision in question. Each item on the scale is phrased as a statement, and respondents can choose from one of five response options: (1) strongly agree, (2) agree, (3) neither agree or disagree, (4) disagree, or (5) strongly disagree. Total scores range from 0 (no conflict) to 100 points (extreme conflict). Scores of 25 or lower are associated with follow-through on decisions, while scores that exceed 38 are associated with delay in decision making [13]. This scale is written at a grade eight reading level and may be difficult for individuals with low literacy levels to interpret and respond to [1, 8]. It has been validated in several languages [14–19] and used in a broad range of medical specialties [20].

#### Low literacy DCS

The low literacy version of the DCS is a 10-item instrument comprised of four subscales: (1) informed, (2) values clarity, (3) support, and (4) uncertainty. Each item is phrased as a question, and respondents can choose from one of three response options: (1) yes, (2) no, or (3) unsure. Total scores range from 0 (no decisional conflict) to 100 points (extreme decisional conflict). This scale was developed for use with individuals with limited reading and response skills [8].

### Psychometric properties of the decisional conflict scales

The original DCS has been established as a reliable and valid instrument appropriate for use in a variety of clinical specialties. Studies of internal consistency have reported Cronbach’s alphas ranging from good to excellent [2, 21–23]. Only one study, to our knowledge, investigated the psychometric properties of the low literacy version of the DCS; in this study, Cronbach’s alpha for the total scale was ≥0.83 [24]. Most efforts to establish construct validity have been successful [2, 22, 23]. These findings suggest that the decisional conflict scales, as a whole, reliably measure the construct of decisional conflict in a way that is congruent with its conceptual definitions [25].

Despite the strengths of the DCS, evidence in support of the reliability and validity of each of its subscales is less clear. Specifically, psychometric testing of the support subscale has yielded inconsistent results. Although the support subscale is theoretically comprised of factors that contribute to uncertainty, the support subscale was poorly correlated with the uncertainty subscale in two instances [21, 24]. Two investigative teams conducted factor analyses and found that items on the support subscale either loaded onto different factors or did not load at all [2, 24]. In the single study that examined the psychometric properties of the low literacy DCS, Cronbach’s alphas for the support subscale were low and ranged from 0.468 to 0.596 [24]. These findings raise the question of whether these items adequately measure support as it is defined conceptually.

The psychometric properties of the low literacy version of the DCS have not been evaluated to the same extent as those of the original version. Further psychometric evaluation of the low literacy version of the DCS is necessary given its reduced number of items and response options. The remainder of this paper is therefore focused on our efforts to assess the properties of the original and low literacy DCS scales and subscales using a novel approach.

### Item response theory

Prior evaluations of the decisional conflict scales have relied solely on classical measurement theory (CMT) to assess the scales’ psychometric properties. CMT focuses on the performance of a scale as a whole rather than on the performance of individual items [25]. It assumes that items within a test are sampled at random from a domain of relevant items. Reliability is seen as a characteristic of the test and of the variance of the latent trait (e.g. decisional conflict) it purports to measure. Items are treated as random replicates of each other and their characteristics, if examined at all, are expressed as correlations with the total test score or as factor loadings on the putative latent variable(s) of interest. Individual items are assumed to provide the same amount of information about the latent trait [26]. As the number of items on the scale increases, usually so does the scale’s measure of internal consistency [25].

Item response theory (IRT) is an alternative to CMT in that it examines the unique relationship of each individual item as well as the whole scale to the latent trait of interest. It permits the researcher to examine the ways in which the precision of an item or scale may differ for individuals possessing different amounts of the latent trait [26, 27]. Unlike CMT analyses, which are sample-dependent, the item parameter estimates derived from IRT are relatively more independent of the sample from which data are collected [28].

IRT encompasses an assortment of mathematical models for binary or categorical outcomes [26]. It mathematically models the relationship between the amount of latent trait possessed by the respondent and the way in which the respondent responds to items on a scale. In this way, IRT analyses determine the characteristics of items and estimate the level of ‘ability’ or ‘trait’ of respondents. This relationship is depicted by an item characteristic curve (ICC), which is a monotonic probability curve that describes the probability (y-axis) of endorsing the item (with dichotomous response categories) for a continuous level of latent trait (x-axis). In the case of items with polytomous response categories (such as a Likert scale), multiple category characteristic curves (CCCs) are used; each CCC depicts the probability of endorsing a single response category.

The three most popular unidimensional IRT models are the one-, two-, and three-parameter logistic models, so named because of the number of item parameters each incorporates. The three possible parameters are difficulty, discrimination, and guessing. For items with dichotomous response categories, difficulty is defined as the value of the latent trait needed for a respondent to have a 50% chance of endorsing the item. For items with polytomous response categories, difficulty is a range of values that is bounded by the first and last points at which the CCCs intersect [29]. In designing an instrument intended to differentiate between all levels of a latent trait, a researcher should try to have items with difficulties spread across the full range of the trait.

Discrimination is represented graphically by the slope of the ICC or the CCC. It describes how fast the probability of selecting a given response will change as the amount of latent trait approaches the item difficulty. In other words, an item with a high discrimination parameter can distinguish better between low and high levels of the latent trait. Very low discrimination values are between 0.01–0.34, low values are between 0.35–0.64, moderate values are between 0.65–1.34, high values are between 1.35–1.69, and very high values are equal to or greater than 1.70 [30].

Susceptibility to guessing is the probability that an individual will select a “correct” response or endorsing an item by guessing [30]. Guessing is represented as a vertical shift of the ICC or CCC curves. The value of this parameter can range from 0 to 1, with values > 0.35 deemed unacceptable. For many patient-reported outcome measures, including decisional conflict, susceptibility to guessing is not considered to be an applicable parameter and is typically not modeled [27].

IRT also provides a measure of the precision of an item in estimating the latent trait [30]. This measure is called information and is represented graphically by the item information function (IIF) and the test information function (TIF), which respectively convey the amount of information provided by each item and the measure as a whole. The Graded Response Model (GRM) [31] is a two-parameter categorical IRT model for scales with ordered response options (e.g. a Likert scale). It models all items in a scale allowing different difficulties for each item and different or equal discrimination parameters across items. A log-likelihood test is usually used to determine whether models with different discrimination parameters are necessary.

## Methods

The aim of this study was to describe the properties of the original (DCS-12) and low literacy (LL DCS-10) Decisional Conflict Scales using techniques associated with Classical Measurement Theory and Item Response Theory. We conducted a secondary analysis of data from two multi-site trials that tested the Personal Patient Profile-Prostate (P3P), a tailored decision aid for men with localized prostate cancer (LPC). The details of these trials, herein referred to as P3P-I and P3P-II, have been reported elsewhere [32, 33]. Briefly, in P3P-I, baseline decisional conflict was measured using the original DCS among men with LPC who were candidates for at least two treatment options and who had not yet begun therapy [32]. The study was approved by the Fred Hutchinson Cancer Research Center/University of Washington Cancer Consortium Review Board and the review board at each site. In P3P-II, baseline decisional conflict was measured using the low literacy DCS among men with LPC who had had no more than one consultation visit, had not made a final care decision, and had not begun active surveillance or received any prostate cancer treatment [33]. The study was approved by the Dana-Farber/Harvard Cancer Center institutional review board and the review board at each site.

In the current study, data from P3P-I were used to analyze the properties of the original DCS. To most effectively compare the original DCS to the low literacy DCS, we excluded data pertaining to the “effective decision” subscale. This subscale is not administered to individuals who have not yet made a decision; herein, we refer to the original DCS that excludes this subscale as the DCS-12. Data from P3P-II were used to analyze the properties of the low literacy DCS, herein referred to as the LL DCS-10. In addition, we analyzed the properties of a hypothetical aggregate version of the DCS-12 in which the original five item response options were aggregated into three response options (strongly agree/agree, neither agree nor disagree, and disagree/strongly disagree). The aggregated version of the DCS-12 was conceptualized for the sole purpose of exploring the effect of reducing the number of response options from five to three, as has been done in LL DCS-10. The aggregate DCS-12 was derived from P3P-I data and scored as the LL DCS-10 is scored, with possible scores ranging 0–100. Cases with complete data on DCS were included in the analysis; no imputation was done for missing data. For all three scales, internal consistency was estimated for the total scale and four subscales by Cronbach’s alphas. Correlations among subscales were evaluated using Pearson’s correlation coefficient. Each of the three scales were modelled using the Graded Response Model (GRM) [31]. Two models were fitted: the constrained GRM, which assumes equal discrimination parameters across items; and the unconstrained GRM, which permits different discrimination parameters across items. The likelihood ratio test was used for model selection. Category characteristic curves (CCC), item information functions (IIF), and test information functions (TIF) were plotted using the model selected for each scale.

## Results

### P3P-I trial

Baseline and clinical characteristics of the 494 men who participated in P3P-I [32] are provided in Table 1. A total of 21 cases with missing data on any item were removed from the analysis, leaving 473 cases in the analysis.

**Table 1:** Baseline and clinical characteristics by study

	Study			
	P3P-I (*n* = 494)		P3P –II (*n* = 392)	
	*N*	Percent	*N*	Percent
Age				
< 60 years	159	32.2	125	31.9
> 60 years	335	67.8	267	68.1
Education				
> High school	280	56.9	310	79.1
≤ High school	214	43.3	77	19.6
Missing/Unknown	0	0	5	1.3
Marital/partner status				
Married/partnered	378	76.5	280	71.4
Not married/partnered	115	23.3	109	27.8
Missing/unknown	1	0.2	3	0.8
Race				
Black	67	13.6	113	28.8
White Hispanic	7	1.4	12	3.1
White non-Hispanic	400	81.0	240	61.2
Others	20	4.1	27	6.9
Working Status				
Not working	2	0.4	162	41.3
Working	273	55.3	225	57.4
Missing/unknown	219	44.4	5	1.3
Income				
< 39,999 (35,000^a^)	110	22.2	97	24.7
> 39,999 (35,000^a^)		73.7	258	65.8
Missing/Unknown	20	4	37	9.4
Web as an information source				
No	153	31.0	76	19.4
Yes	341	69.0	282	71.9
Missing/Unknown	0	0	34	8.7
D’Amico risk classification				
High			60	15.3
Intermediate			194	49.5
Low			135	34.4
Missing/unknown			3	0.8

^a^An income threshold of 35,000 was used in P3P-I

#### DCS-12

Means and standard deviations for the DCS-12 total and subscale scores are provided in Table 2. Cronbach’s alphas for the DCS-12 total scale and three of four subscales were ≥ 0.85; for the support subscale, Cronbach’s alpha was 0.57 (Table 2). Correlations across 4 subscales ranged from 0.44–0.71 and were significant at the *p* < 0.001 level.

**Table 2:** Means, standard deviations (SD), and Cronbach’s alphas for total scales and subscales

Subscale	Mean (SD)			Cronbach’s alpha		
	DCS-12	Aggregated DCS-12	LL DCS-10	DCS-12	Aggregated DCS-12	LL DCS-10
DCS Total				0.91	0.89	0.88
Uncertainty	52.3 (26.9)	52.7 (39.1)	62.8 (35.5)	0.90	0.89	0.89
Informed	38.2 (23.8)	31.4 (35.6)	46.9 (32.8)	0.85	0.88	0.89
Value Clarity	34.9 (21.2)	25.2 (31.4)	40.8 (36.8)	0.88	0.86	0.88
Support	29.8 (16.4)	21.6 (20.6)	28.1 (21.4)	0.57	0.40	0.34

**Fig. 1:**
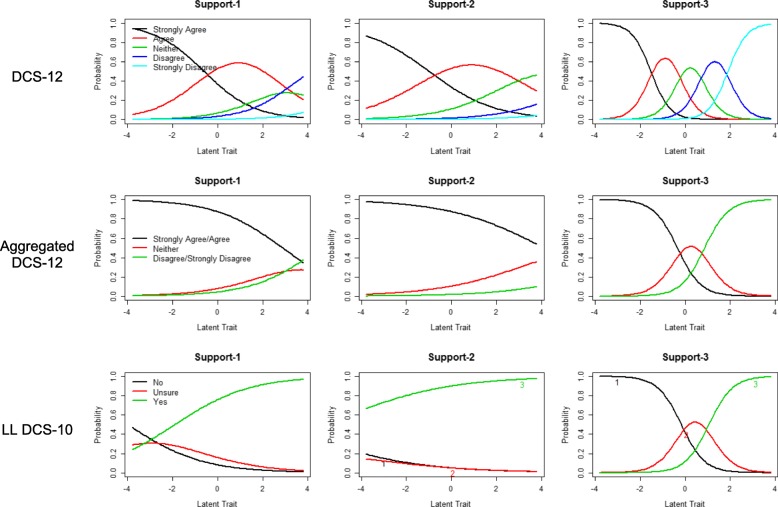
Category characteristic curves for support subscale items on the DCS-12, aggregated DCS-12, and LL DCS-10. Probability (y-axis) represents the probability that a respondent will select a response option, given the respondent’s latent trait value (x-axis). In this analysis, decisional conflict is the latent trait being measured. For the DCS-12, response options are as follows: 0 (strongly agree), 1 (agree), 2 (neither agree nor disagree), 3 (disagree), 4 (strongly disagree) For the aggregated DCS-12, response options are as follows: 0–1 (strongly agree/agree), 2 (neither agree nor disagree), 3–4 (disagree/strongly disagree). For the LL DCS-10, response options are yes, no, and unsure

**Fig. 2:**
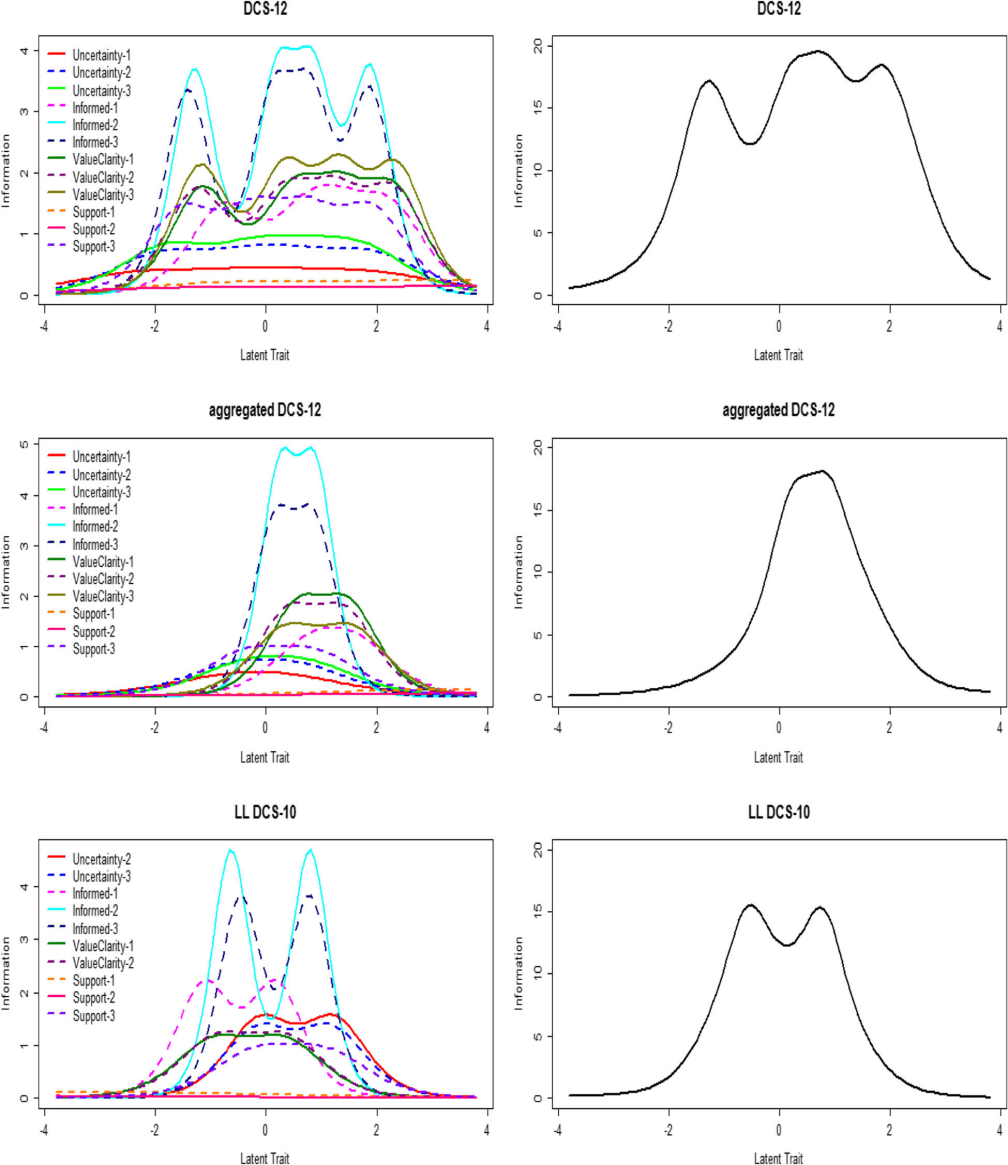
Item information functions and test information functions for the DCS-12, aggregated DCS-12, and LL DCS-10. Information (y-axis) represents the precision with which a respondent’s latent trait value (x-axis) can be estimated. Higher information indicates greater precision. In this analysis, decisional conflict is the latent trait being measured

The unconstrained GRM was selected (*p* < 0.001) as a better fit. Item difficulty ranged from approximately − 2 to 2 for most items. Two notable exceptions are the items support-1 and support-2, the difficulty of which ranged from approximately − 1 to > 4. The CCCs for the items on the support subscale are depicted in Fig. 1. Item discrimination was high to very high for most items, with discrimination values ranging from 1.6 to 3.8. Exceptions included the items uncertainty-1, support-1, and support-2, which had moderate discrimination values of 1.19, 0.89, and 0.68, respectively. The IIFs (Fig. 2) demonstrate that the least amount of information is provided by items support-2, support-1, and uncertainty-1, in ascending order. Items informed-2 and informed-3 provided the most information. As depicted by the TIF (Fig. 2), the DCS-12 provides the most test information (range 13–20) across a wide range of latent trait values from − 2 to 3 and provides at least some information for nearly the entire range of latent trait values. The DCS-12 also demonstrates good estimation precision with high test information across a wide range of latent traits from − 2 to 3.

#### Aggregated DCS-12

Means and standard deviations for the aggregated DCS-12 total and subscale scores are provided in Table 2. Cronbach’s alphas for the aggregated DCS-12 total scale and three of four subscales were ≥ 0.86; for the support subscale, Cronbach’s alpha was 0.40 (Table 2). Correlations across 4 subscales ranged from 0.42–0.62 and were significant at the *p* < 0.001 level.

The unconstrained GRM was selected (*p* < 0.001) as a better fit. Most item difficulties fell in the range of approximately − 0.5 to 1.5. Again, the items support-1 and support-2 were exceptions, with difficulties ranging from approximately 2.8 to > 4. The CCCs for the items on the support subscale are depicted in Fig. 1. Item discrimination was high to very high for most items, with discrimination values ranging from 1.5 to 4.3. Discrimination was moderate for the items uncertainty-1 (1.3) and support-1 (0.68), while discrimination was low for the item support-2 (0.47). As is the case for the non-aggregated DCS-12, the IIFs (Fig. 2) demonstrate that the least amount of information is provided by the items support-2 and support-1, while informed-2 and informed-3 provided the most information. The TIF (Fig. 2) indicates that the aggregated DCS-12 provides the most test information (range 8–18) when latent trait values range from approximately − 0.5 to 2 and provides little information for latent trait values below − 1.

### P3P-II trial

Baseline and clinical characteristics of the 392 men who participated in P3P-II [33] are provided in Table 1. A total of 365 cases had complete DCS data and were used in the following analysis.

#### LL DCS-10

Means and standard deviations for the LL DCS-10 total and subscale scores are provided in Table 2. Cronbach’s alphas for the LL DCS-10 total scale and three of four subscales were ≥ 0.88; for the support subscale, Cronbach’s alpha was 0.34 (see Table 2). Correlations across 4 subscales ranged from 0.44–0.68 and were significant at the *p* < 0.001 level.

The unconstrained GRM was selected (*p* < 0.001) as a better fit. The difficulty of most items fell in the range of approximately − 1 to 1. Again, the items support-1 and support-2 had anomalous difficulty parameters that ranged from − 4.0 to − 1.9 for support-1 and -7.4 to − 5.5 for support-2. The CCCs for the items on the support subscale are depicted in Fig. 1. Item discrimination was generally high to very high, with discrimination values ranging from 1.9 to 4.3 for most items. However, discrimination was low for the items support-1 (0.6) and support-2 (0.4). The IIFs (Fig. 2) demonstrate that items support-2 and support-1 provided the least amount of information, while items informed-2 and informed-3 provided the most information. As depicted by the TIF (Fig. 2), the LL DCS-10 provides the most test information (range 5–15) when latent trait values range from − 1.5 to 1.5 but provides nearly little or no information for latent trait values outside of that range.

Category characteristic curves for items on all three scales are provided in Additional file 3.

## Discussion

This was the first known study in which an IRT approach was used to describe the properties of the original and low-literacy DCS. As such, the findings of this study provide new information regarding the relationship between the items on these scales and decisional conflict as a latent trait. Moreover, the findings from this study provide insight into the ways in which the precision of these scales may differ for individuals possessing different amounts of decisional conflict [26, 27].

The results of our CMT-based analyses are consistent with prior research suggesting that while the decisional conflict scales as a whole are reliable, the support subscale may be less so. For each of the three scales that were assessed, the support subscale had markedly lower internal consistency than the other three subscales. This finding raises the question of whether the support subscale measures the same underlying construct as the other subscales. Alternatively, these low alphas may be related to a skewed distribution of support scores towards high decisional support [25].

The results of our IRT-based analyses provide a closer look at the properties of the support subscale. For all three scales, the items support-1 (which asks whether the respondent has “enough support from others to make a choice”) and support-2 (which asks whether the respondent is “choosing without pressure from others”) have difficulty parameters that are extreme relative to those of other items. In our analyses of the aggregated DCS-12 and the LL DCS-10, the response options “strongly agree/agree” (on the aggregated DCS-12) and “yes” (on the LL DCS-10) are the most likely response options across nearly the entire range of latent trait values, suggesting that even very conflicted respondents are likely to report that they have enough support to make a choice and are choosing without pressure from others (Fig. 1). This finding is consistent with a skewed distribution towards high decisional support. For all three scales, these two items discriminate less between respondents with different amounts of decisional conflict than other items. The discrimination parameters for these items were lower on the aggregated DCS-12 and the LL DCS-10 than on the original DCS-12, yet even on the DCS-12, discrimination parameters for these items were substantially lower than for any other items on the scale. Considering these findings, it is perhaps not surprising that the IIFs for all three scales revealed that the items support-1 and support-2 contribute the least amount of information to the test (Fig. 2). The IIF curves for each of these items are relatively flat, indicating that these two items provide relatively little information across the range of potential values of decisional conflict, raising questions about their utility for measuring the scales’ target construct.

The reason that support-1 and support-2 do not perform as well as the other items on the DCS is unclear. The inclusion of items on the DCS that address support and pressure is theoretically sound. According to the ODSF, factors such as knowledge, expectations, values, and decisional conflict represent an individual’s perception of a decision, while factors such as norms, pressure, support, and decision role preference represent the perceptions of important others [11]. Conducting cognitive interviews that focus on the wording of these particular items may provide insight into respondents’ interpretations and responses.

Our IRT analyses suggest that, compared with the aggregated DCS-12 and the LL DCS-10, the original DCS-12 is most capable of discriminating between respondents with high and low amounts of decisional conflict. The items on the DCS-12 comprise a wider range of item difficulties than those on the aggregated DCS-12 or the LL DCS-10, indicating that the original DCS-12 is capable of discriminating between respondents across a wider range of potential decisional conflict values. Similarly, the TIFs (Fig. 2) reveal that the DCS-12 provides more information (higher estimation precision) than the other scales across a wider range of latent trait values. These findings suggest that the tradeoff for a reduced number of response options may be a decline in scale precision, particularly for respondents with high and low decisional conflict. The extent to which the reduction in response options has affected the LL DCS-10’s readability is unclear and is worthy of further study.

The current study was limited by its use of data from two trials with similar, but not identical, samples. Comparison of the DCS-12 and LL DCS-10 in the same sample may provide additional insight.

## Conclusions

The original DCS-12 can discriminate between respondents across a wide range of decisional conflict values. The results of this analysis may be used to guide instrument selection for the evaluation of interventions aimed at reducing decisional conflict. Further research is needed to determine how best to improve the performance of the support subscale and how to meet the needs of low literacy populations without sacrificing precision.

## Additional files

Additional file 1: Decisional conflict defining characteristics and related factors. This table presents the defining characteristics of decisional conflict and its related factors as defined by NANDA International [10]. (DOCX 13 kb)

Additional file 2: Subscales and associated items on the DCS-12 and LL DCS-10. This table provides an overview of the four subscales and associated items for the DCS-12 and LL DCS-10 as developed by O’Connor [1]. This information is included to assist the reader in interpreting the results of the current study; for full details regarding the DCS-12 and LL DCS-10, the reader is referred to the original content developed by O’Connor [1]. (DOCX 14 kb)

Additional file 3: Category characteristic curves for all subscales on the original DCS-12, aggregated DCS-12, and LL DCS-10. This figure displays the category characteristic curves for items on each subscale of the original DCS-12, aggregated DCS-12, and LL DCS-10. (DOCX 1243 kb)

## Data Availability

The datasets generated and analysed during the current study are not publicly available due to institutional review board regulations, but are available from the corresponding author on reasonable request.

## Abbreviations

CCC: Category characteristic curve
CMT: Classical measurement theory
DCS: Decisional Conflict Scale
DCS-12: The 12-item original Decisional Conflict Scale
GRM: Graded response modeling
ICC: Item characteristic curve
IIF: Item information function
IRT: Item response theory
LL DCS-10: The 10-item low literacy Decisional Conflict Scale
LPC: Localized prostate cancer
NANDA-I: North American Nursing Diagnosis Association - International
ODSF: Ottawa Decision Support Framework
P3P: Personal Patient Profile-Prostate
TIF: Test information function
