# Supplementary material for: Item response theory analysis and properties of decisional conflict scales: findings from two multi-site trials of men with localized prostate cancer
Source: BMC Med Inform Decis Mak. 2019 Jul 4;19:124. doi: 10.1186/s12911-019-0853-5 (PMC6610903; doi:10.1186/s12911-019-0853-5)
Supplement: Supplementary file 1 — Decisional conflict defining characteristics and related factors. This table presents the defining characteristics of decisional conflict and its related factors as defined by NANDA International [10]. (DOCX 13 kb) [file 12911_2019_853_MOESM1_ESM.docx]

**Additional File 1.** Decisional conflict defining characteristics and related factors

| Defining Characteristics | Related Factors |
| --- | --- |
| Delay in decision-making | Conflict with moral obligation |
| Distress while attempting a decision | Conflicting information sources |
| Physical signs of distress (e.g. increase in heart rate, restlessness) | Inexperience with decision-making |
| Physical sign of tension | Insufficient information |
| Questioning of moral principle while attempting  a decision | Insufficient support system |
| Questioning of moral rule while attempting  a decision | Interference in decision-making |
| Questioning of moral values while attempting  a decision | Moral principle supports mutually inconsistent actions |
| Questioning of personal beliefs while attempting  a decision | Moral rule supports mutually inconsistent actions |
| Questioning of personal values while attempting  a decision | Moral value supports mutually inconsistent actions |
| Recognizes undesired consequences of actions being considered | Perceived threat to value system |
| Self-focused | Unclear personal beliefs |
| Uncertainty about choices | Unclear personal values |
| Vacillating among choices |  |

*Note.* Adapted from NANDA International, 2014, p. 364 [1].

[1] NANDA International, Nursing Diagnoses: Definitions and Classification 2015-2017, 10 ed., Wiley Blackwell, Hoboken, NJ, 2014.
