# Supplementary material for: Item response theory analysis and properties of decisional conflict scales: findings from two multi-site trials of men with localized prostate cancer
Source: BMC Med Inform Decis Mak. 2019 Jul 4;19:124. doi: 10.1186/s12911-019-0853-5 (PMC6610903; doi:10.1186/s12911-019-0853-5)
Supplement: Supplementary file 2 — Subscales and associated items on the DCS-12 and LL DCS-10. This table provides an overview of the four subscales and associated items for the DCS-12 and LL DCS-10 as developed by O’Connor [1]. This information is included to assist the reader in interpreting the results of the current study; for full details regarding the DCS-12 and LL DCS-10, the reader is referred to the original content developed by O’Connor [1]. (DOCX 14 kb) [file 12911_2019_853_MOESM2_ESM.docx]

**Additional File 2.** Subscales and associated items on the DCS-12 and LL DCS-10

| **Subscale** | **Item** | **DCS-12** | **LL DCS-10** |
| --- | --- | --- | --- |
| Informed | 1 | I know which options are available to me. | Do you know which options are available to you? |
| Informed | 2 | I know the benefits of each option. | Do you know the benefits of each option? |
| Informed | 3 | I know the risks and side effects of each option. | Do you know the risks and side effects of each option? |
| Values Clarity | 1 | I am clear about which benefits matter most to me. | Are you clear about which benefits matter most to you? |
| Values Clarity | 2 | I am clear about which risks and side effects matter most. | Are you clear about which risks and side effects matter most to you? |
| Values Clarity | 3 | I am clear about which is more important to me (the benefits or the risks and side effects). | N/A |
| Support | 1 | I have enough support from others to make a choice. | Do you have enough support from others to make a choice? |
| Support | 2 | I am choosing without pressure from others. | Are you choosing without pressure from others? |
| Support | 3 | I have enough advice to make a choice. | Do you have enough advice to make a choice? |
| Uncertainty | 1 | I am clear about the best choice for me. | Are you clear about the best choice for you? |
| Uncertainty | 2 | I feel sure about what to choose. | Do you feel sure about what to choose? |
| Uncertainty | 3 | This decision is easy for me to make. | N/A |

*Note.* Adapted from O’Connor, 2010 [1].

[1] A. O'Connor, User Manual - Decisional Conflict Scale, Ottawa Hospital Research Institute, Ottawa, ON, 2010.
