# Supplementary material for: Item response theory analysis and properties of decisional conflict scales: findings from two multi-site trials of men with localized prostate cancer
Source: BMC Med Inform Decis Mak. 2019 Jul 4;19:124. doi: 10.1186/s12911-019-0853-5 (PMC6610903; doi:10.1186/s12911-019-0853-5)
Supplement: Supplementary file 3 — Category characteristic curves for all subscales on the original DCS-12, aggregated DCS-12, and LL DCS-10. This figure displays the category characteristic curves for items on each subscale of the original DCS-12, aggregated DCS-12, and LL DCS-10. (DOCX 1243 kb) [file 12911_2019_853_MOESM3_ESM.docx]

**Additional File 3.** Category characteristic curves for all subscales on the original DCS-12, aggregated DCS-12, and LL DCS-10.

| **DCS-12** | 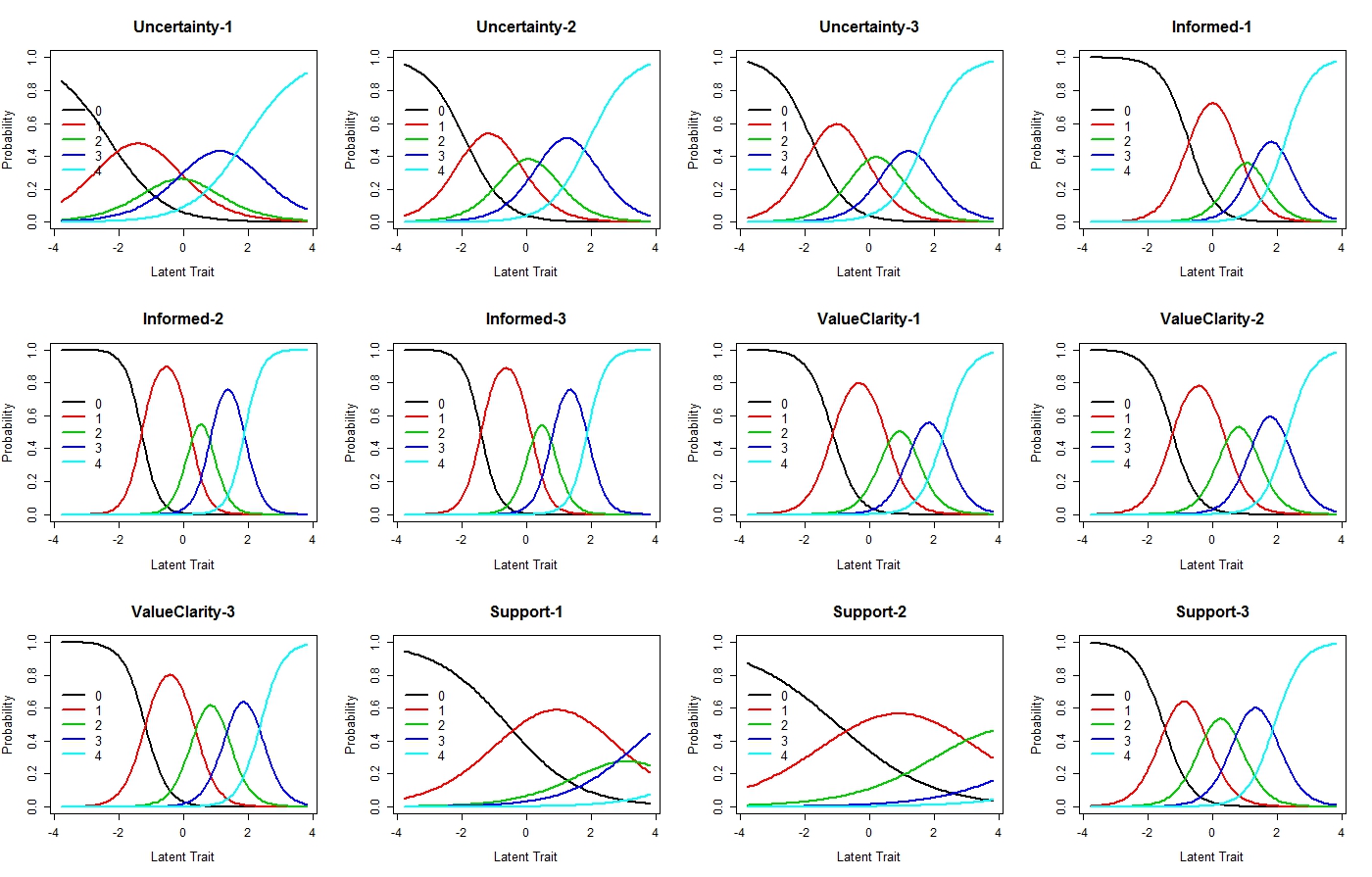 |
| --- | --- |
| **Aggregated DCS-12** | 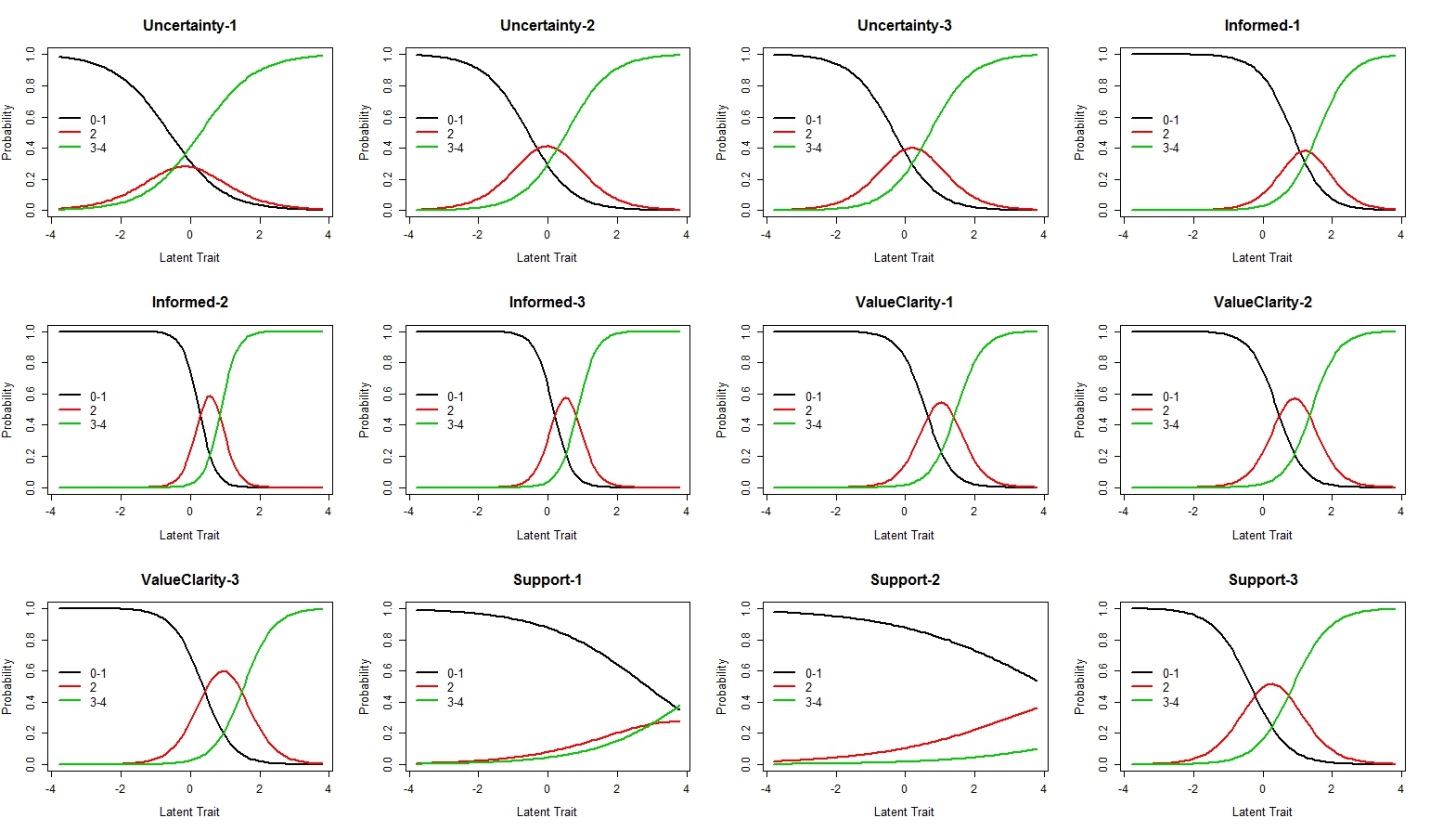 |
| **LL DCS-10** | 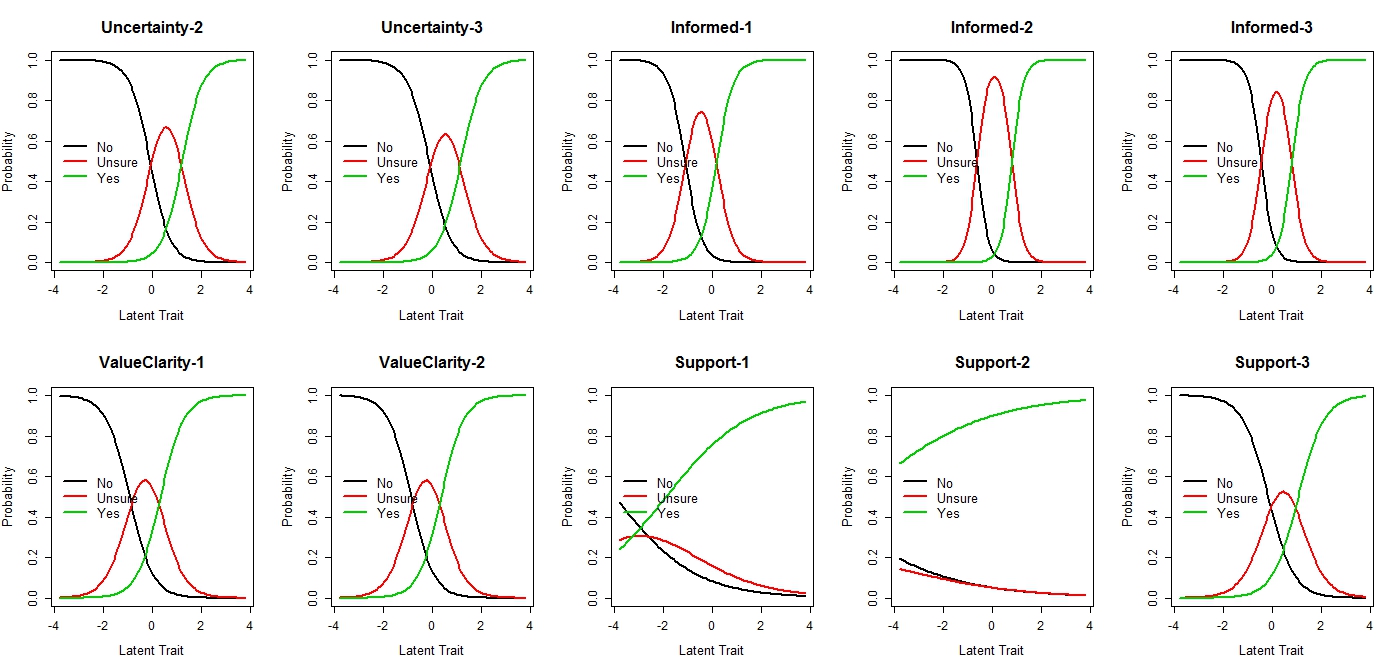 |
